# Behavioral Economics and Parent Participation in an Evidence-Based Parenting Program at Scale

**DOI:** 10.1007/s11121-021-01249-0

**Published:** 2021-05-20

**Authors:** Zoelene Hill, Michelle Spiegel, Lisa Gennetian, Kai-Ama Hamer, Laurie Brotman, Spring Dawson-McClure

**Affiliations:** 1 New York Academy of Medicine , NY 10029 New York, United States; 2 University of California , CA 92697 Irvine, United States; 3 Duke University , NC 27708 Durham, United States; 4 NYU Grossman School of Medicine , NY 10016 New York, United States; 5 NYU Grossman School of Medicine , NY 10016 New York, United States; 6 NYU Grossman School of Medicine , NY 10016 New York, United States

**Keywords:** BE, Parent engagement, Low-income, Parenting program, Text messaging

## Abstract

Evidence-based and culturally relevant parenting programs strengthen adults’ capacity to support children’s health and development. Optimizing parent participation in programs implemented at scale is a prevailing challenge. Our collaborative team of program developers, implementers, and researchers applied insights from the field of behavioral economics (BE) to support parent participation in ParentCorps—a family-centered program delivered as an enhancement to pre-kindergarten—as it scaled in a large urban school district. We designed a bundle of BE-infused parent outreach materials and successfully showed their feasibility in site-level randomized pilot implementation. The site-level study did not show a statistically significant impact on family attendance. A sub-study with a family-level randomization design showed that varying the delivery time of BE-infused digital outreach significantly increased the likelihood of families attending the parenting program. Lessons on the potential value of a BE-infused approach to support outreach and engagement in parenting programs are discussed in the context of scaling up efforts.

**Supplementary Information:**

The online version contains supplementary material available at 10.1007/s11121-021-01249-0.

Supporting caregivers as children transition to preschool is increasingly recognized as a strategy to support children’s development and to reduce socioeconomic and racial/ethnic disparities therein (Brotman et al., 2011; Dawson-McClure et al., 2017; Gross et al., 2009; Reid et al., 2001). Group-based, culturally relevant parenting programs have been shown to increase warm, nurturing parent–child interactions, consistent discipline, and involvement in children’s learning; decrease parent stress; and have long-lasting favorable impacts on children’s academic and health outcomes (Brotman et al., 2016; Dawson-McClure et al., 2015; Grindal et al., 2016; Kaminski et al., 2008). The promise of parent-focused evidence-based interventions (EBIs) hinges on programs being able to optimally engage families at scale (Sanders, 2008).

However, optimizing parent attendance in scaled programs remains challenging (Bumbarger & Perkins, 2008; Dawson-McClure et al., 2017; Fagan et al., 2009). A population-level trial of Triple P in the southeastern USA indicated that only 1% of parents attended the level 4 group-based program (Prinz et al., 2009). Similarly, 5% of parents attended a parenting program in the Communities That Care trial (Fagan et al., 2009). A study of an evidence-based parenting program delivered at scale through family courts found that more than half of parents expressed an intent to participate or interest in learning more, but only 10% actually attended one or more sessions (Wolchik et al., 2009). Further, empirical investigations of prevention or school-based programs show lower overall levels of engagement, as measured by program attendance, from families with low-income (Baker et al., 2011; Whittaker & Cowley, 2012); racial/ethnic minority groups (Baker et al., 2011); parents with low levels of self-efficacy (Chacko et al., 2017); families with low levels of social support (Baker et al., 2011); and families with high levels of psychological stress (Minney et al., 2015). Achieving attendance at levels consistent with program goals remains a conundrum in part because parent and caregiver interest and intentions do not translate to subsequent follow-through (Wolchik et al., 2009). A task force formed to address the limited reach of EBIs through public systems emphasized the importance of partnerships between EBI developers, practitioners, and policymakers to optimize EBIs based on deep understanding of the implementing system and the end users (Fagan et al., 2019).

We describe a collaborative effort to apply a behavioral economics (BE) lens to optimize outreach and family engagement in parenting programs at scale in public systems. Behavioral economics is a multi-disciplinary framework that draws on theories and concepts from social psychology, psychology, and economics to understand how contexts can affect decision-making. It complements other frameworks by considering parents’ in-the-moment decision-making in the context of their circumstances, including those shaped by poverty and racism. In this study, we document how BE concepts were translated into concrete outreach materials and strategies for use in a parenting program as it scaled up in a large urban school district, and we measure the influence of testable components of the BE-infused strategies on parent participation.

The BE perspective contributes to conceptual and empirical work on family engagement in evidence-based parenting programs in two ways. First, it situates parents as key decision-makers who are influenced by their social, psychological, economic, and historical contexts. Second, it views family engagement as a complex and interrelated chain of decisions beginning with expressions of initial interest, subsequent formulation of intentions to participate (or not), follow-through on intentions to participate (or not), and application of new knowledge and skills at home (or not). Thus, BE moves beyond the focus on one big decision such as “did a family attend the program?” to an understanding of the sequence of interrelated decisions that collectively contribute to observed behavior. It expands on existing theories by challenging the idea that parents consistently act in ways that reflect carefully constructed intentions or pre-existing values about a behavior (e.g., participating in a parenting program) absent their circumstances and historical experiences.

## Efficacy of Existing Frameworks of Participation in Parenting Programs

Theoretical frameworks like the theory of planned behavior (TPB) (Ajzen, 1991) and the health beliefs model (HBM; Rosenstock, 1974; Salari & Filus, 2017) describe how preexisting beliefs, attitudes, and values affect intentions and behaviors. The TPB posits that attitudes (i.e., personal opinions about the behavior), subjective norms (i.e., perceived social pressure to perform the behavior), and perceived control (i.e., perceived ease or difficulty of performing a behavior) determine intentions and, subsequently, behavior (Ajzen, 1991). The HBM posits that behavior depends on the value an individual places on a goal and the individual’s sense that a behavior will lead to achieving that goal (Janz & Becker, 1984). These frameworks complement conventional economic-motivated approaches of encouraging attendance by reducing transaction and related costs (e.g., providing transportation, child care, and food) or by providing financial incentives or rewards for attendance (i.e., covering opportunity cost, in the language of economics). Researchers have used the TPB and HBM frameworks to measure predictors of engagement and to recommend parent engagement strategies (e.g., newsletters to influence parents’ perceptions (Randolph et al., 2009) and norm-based strategies (Bracke & Corts, 2012). Winslow et al. (2016) harnessed insights from TPB to improve parent engagement by including motivational interviewing and goal setting to increase parents’ follow-through with intentions.

Although existing frameworks suggest useful ways to achieve desired engagement with programs, gaps remain. Some strategies are low-cost and scalable (i.e., giving families program information via a newsletter). Other strategies require more resources that may be feasible to offer on a large scale (i.e., child care cost subsidies or individualized motivational interviewing). Furthermore, existing frameworks offer little guidance on the role of timing of outreach strategies at key decision-making points.

## Contribution of Behavioral Economics to Participation in Parenting Programs

BE starts from an understanding of human decision-making as a function of evaluating costs and benefits (as proposed in conventional economics) coupled with the way humans are influenced by their psychological biases and contexts. BE expands on existing theories by challenging the idea that parents consistently act in ways that reflect carefully constructed intentions or pre-existing values about a behavior and, further, do so in a context-free way. As such, BE turns a lens on how current and prior experiences with poverty, financial instability, structural inequality, and/or discrimination can shape or undermine intentions (Gennetian & Shafir, 2015; Mullainathan & Shafir, 2013). Poverty, as a circumstance of scarce and unstable economic resources coupled with repeated racially or ethnically marginalizing experiences, is especially draining on cognitive resources (Gennetian & Shafir, 2015). First, it is exhausting to engage in cost–benefit trade-offs and calculations to evaluate future choices and behaviors, and thus, the easier choice might prevail even if it is not the preferred choice or the choice known to have better long-term prospects. Second, the consequences of this cognitive (decision making) pressure under circumstances of scarce and unstable resources can be more damaging than for those with economic cushions who can recover, for example, from a financial error.

BE considers the role of cognitive resources, such as attention, as scarce and finite, similar to the conventional economic ideas of money and time as resources. Thus, heuristics or other patterns of information, such as those presented in society or by peers, are often relied upon to inform decisions implicitly or explicitly (Baddeley & Hitch, 1974; Valcke, 2002). The influences of such psychological resources and factors can sometimes conflict with or overcome the influences of targeted education or information-based strategies, particularly if they are complex and difficult to digest. The implications of finite cognitive resources are that allocation of such resources to one aspect of family life (e.g., immediate demands like paying rent) may drain cognitive resources for other aspects of family life (e.g., reading or playing with a child (Gennetian et al., 2016) or attending a parenting program). Thus, how parents make sense of information or situations might result in behaviors that deviate from values or intentions.

BE conceptualizes decision-making as consisting of fine-grained moments, such as picking up or deciding to read a brochure about a program, that have spiraling effects on larger decisions typically observed as behavior change. All of these micro-decisions—when combined—contribute to the goal of program attendance and, further, shifts in daily practice based on program learnings. BE focuses on these decision-making junctures with the goal of keeping parents on the ramp to attendance. Lastly, the BE framework offers insights on strategies that can be feasibly implemented within existing program infrastructure in low-cost and scalable ways. Typically called “nudges,” these BE-informed enhancements take the perspective of choice architecture, that is, whether programs are implemented in ways that facilitate or interfere with decision-making intentions (Thaler & Sunstein, 2008).

Recent randomized controlled trials have shown the effectiveness of BE strategies for increasing parent attendance and engagement in a variety of early childhood programs (Gennetian, in press). Researchers used BE strategies such as child-friendly planners, reminders, and personalized invitations to increase Head Start parents’ attendance in a school readiness program and to increase parents’ time spent with children on educational activities at home (Gennetian et al., 2019). Another set of researchers used the BE strategies of reminders, goal setting, and social rewards to increase the time parents spent reading to their children (Mayer et al., 2019). An experiment testing the behavioral concept of default options found that enrolling mothers in a text message-based informational program and providing the option of opting out led to significantly more mothers receiving information, compared with the default of requiring mothers to self-enroll in the program (Gennetian et al., 2020). A survey-based experiment showed that written self-affirmation exercises increased parents’ self-concept and their interest in parenting support programs, especially among parents with a high fear of judgment (Hill et al., 2020). Together, these studies provide increasing evidence of the potential of BE-informed strategies to support ongoing parent outreach efforts and optimize participation in programs.

## ParentCorps: an Opportunity to Pilot BE Insights as a Parenting Program Scales Up

ParentCorps is an enhancement of publicly funded pre-K programs to bolster parent and teacher capacity to support children’s development in the face of early childhood adversity, scaled up as part of New York City’s Pre-K for All starting in 2016 (Brotman et al., 2021). ParentCorps includes multiple components—a 14-week parenting program, which is the focus of this paper, as well as professional development for pre-K teachers and staff and a social-emotional learning program for pre-K classrooms. ParentCorps outreach begins at the start of the academic year with a variety of written materials including flyers, posters, and brochures. Pre-K teachers and family support staff host welcome events and strive to personally invite each pre-K parent. ParentCorps provides meals and small incentives (i.e., raffle tickets for gift cards), and when the program is held in the evenings, parallel programs are offered for children (i.e., social-emotional learning for pre-K children, arts activities for siblings). Parents are always welcomed regardless of constraints (e.g., arriving late from work), and the invitation extends to all adults in the child’s life (e.g., grandparent, aunt). Throughout the program, parents are encouraged to practice parenting strategies at home while receiving support from the facilitator and peers.

In two randomized controlled trials in pre-K programs attended primarily by Black and Latinx families, ParentCorps strengthened teacher-parent relationships, parent involvement in children’s learning, and use of evidence-based parenting practices (e.g., routines, parent–child play, positive reinforcement, discipline); prevented the development of child behavioral and emotional problems at school; improved academic performance and achievement test scores; and, among higher-risk children, prevented obesity and improved health behaviors (Brotman et al., 2011, 2016; Dawson-McClure et al., 2014). Careful analysis of attendance in the second trial, in which university-based staff and pre-K staff shared responsibility for parent outreach and program implementation, showed that 58% of families enrolled in the pre-K programs attended at least one session, with considerable heterogeneity across schools (range, 44% to 75% of parents attending at least one session). Attendance increased from 50% in the first year of implementation to 65% in the fourth year. Parent attendance was not predicted by ethnicity, neighborhood poverty, or baseline parenting or child behavior (Dawson-McClure et al., 2014), indicating that a wide spectrum of families engaged.

In moving from efficacy and effectiveness trials that were conducted in school-based pre-K settings to scaling up in schools and center-based pre-Ks throughout the city, ParentCorps aimed to support pre-K staff in implementing the program with comparable success in family engagement. This required revisiting materials and strategies to be user-friendly and streamlined so that pre-K staff could feasibly take on responsibility for parent outreach as part of their full-time jobs. Further, the revision of the outreach materials was an opportunity to codify the program’s approach to building respectful relationships with parents, not assuming that all parents should prioritize participation in the program, but based on the belief that all parents deserve to be offered the opportunity to participate in ways that affirm their worth and support their autonomy.

## Current Study

This study is a partnership between scholars with expertise in behavioral economics and ParentCorps developers and coaches who support program implementation by pre-K staff. The first aim was to document how BE concepts could feasibly be translated into concrete outreach materials and strategies for ParentCorps as it scaled up in a large urban school district. The second aim was to examine how testable components of the BE-infused outreach strategies influenced parents’ participation in the program, with the hypothesis that parents receiving BE-infused outreach would on average have higher levels of program attendance.

### Translation of BE Concepts Into ParentCorps Outreach Materials

The process of developing BE-infused outreach materials for ParentCorps began with a yearlong collaboration between researchers and ParentCorps developers and coaches. Informed by previous literature on parent engagement, researchers conducted semi-structured interviews and focus groups with parents who participated in ParentCorps to understand their perspectives on the benefits of the program and the contexts that may promote or hinder other parents’ participation. Researchers attended ParentCorps trainings with pre-K staff preparing for implementation to understand the essential values and practices in conducting parent outreach and facilitating the program. Researchers and ParentCorps developers and coaches also had weekly calls to discuss existing ParentCorps outreach, BE principles that could apply to parents’ attendance, and how proposed BE-infused materials could fit into ParentCorps’ outreach strategy.

**Fig. 1:**
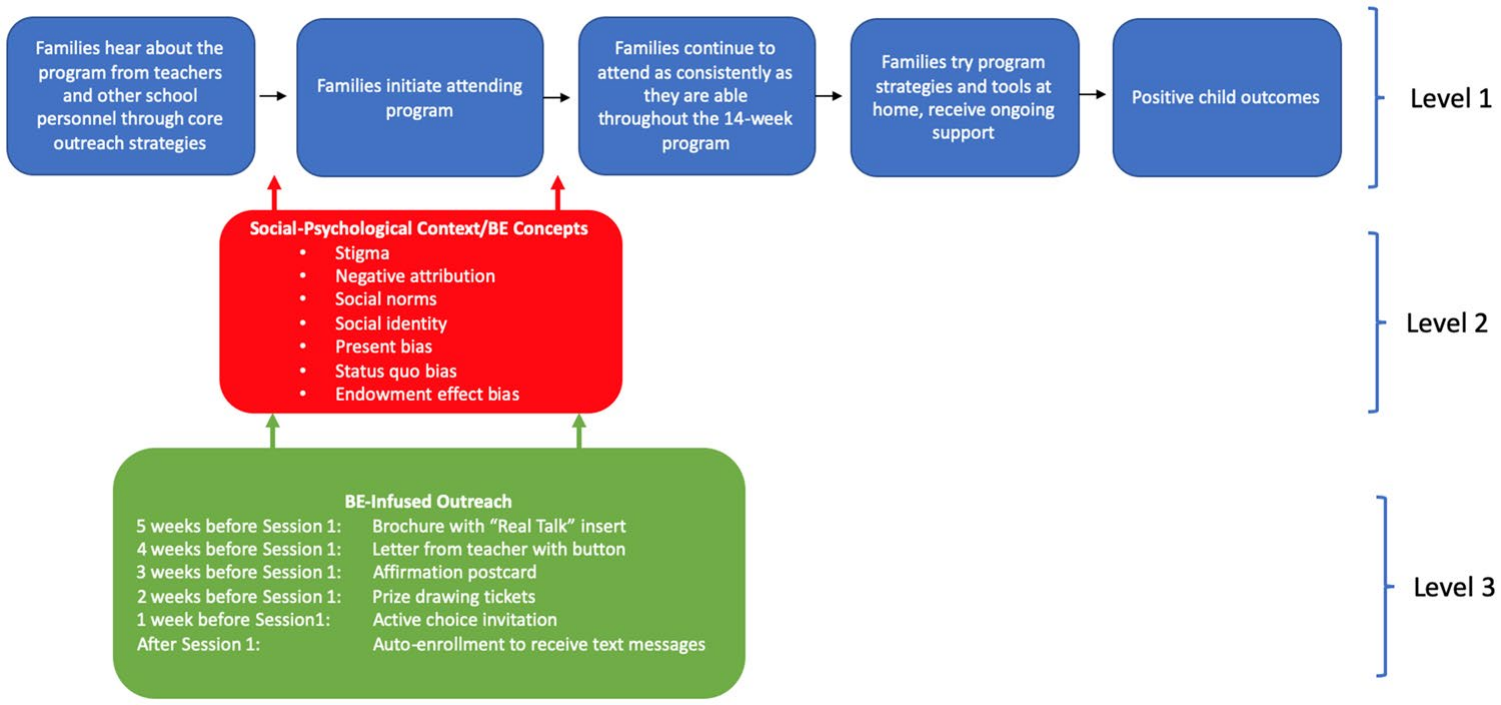
BE-infused program theory of change. *Note.* BE = behavioral economics. Additional program features at level 1 include outreach to families continuing through session 5, reminder phone calls made by teachers and other familiar school personnel to parents throughout the program, meals, and small incentives (i.e., raffle tickets for gift cards), and child care when the program is offered in the evenings. For level 3, within BE-infused outreach sites, families were individually randomized to receive text messages at varying times or the same time each week. See Online Resource Table A for a timeline

One of the first fundamental steps of our collaboration was to develop a BE-informed theory of change (Fig. 1). The theory of change guided us toward decision-making junctures wherein specific psychological contexts (Fig. 1, level 2) could act as bottlenecks slowing or interrupting progress toward the presumed pipeline of behaviors toward achieving positive outcomes. Our identification of psychological contexts likely to influence parents’ decision making was informed by previous literature on parent engagement, and semi-structured interviews and focus groups with ParentCorps parents, developers, and coaches. The identified decision-making junctures served as key points to implement BE-infused materials (Fig. 1, level 3) to facilitate families’ participation.

Several specific psychological contexts were identified as potential bottlenecks (Fig. 1, level 2). *Stigma* and parents’ fear of judgment by others—the belief that participation in a given activity may taint or discount a salient aspect of one’s identity—is posited to interfere with participation in parenting programs (Hall et al., 2014; Mytton et al., 2014). As an example, parents may experience a spoiled identity if they or others believe that parenting programs are for “bad” parents. Parents who receive materials about parenting programs may construe the invitation as a signal that others believe they are a “bad” parent (White & Wellington, 2009). This *negative attribution*, whereby individuals perceive negative intent from others’ motives can be particularly heightened for parents of color, given the commonly held stereotypes that Black and brown parents are uncaring, or ill-equipped to raise successful children (Marchand-Reilly et al., 2019).

*Social norms*, that is, unwritten codes of conduct that guide social interaction in communities (Chung & Rimal, 2016), can shape participation in parenting programs. *Social identity* is closely related to social image, and thus parents’ actions may be influenced by a sense of social belonging or by social pressure. Making a social norm salient—directly through advertisement of the norm or indirectly through images that represent normed behavior, such as a smiley face—has been shown to increase eco-friendly behavior, improve health-related habits, and facilitate cooperation (Cialdini, 2007; Ostrom, 2000).

Individual biases also affect decision-making. *Present bias* places a stronger value on current benefits with large undervaluing (discounting) of future benefits (Mischel et al., 2011). Benefits associated with warm, nurturing parenting are long reaching and difficult to quantify. Making those long-term benefits concrete or increasing the salience of the short-term behavior that contributes to them can be effective in certain early literacy interventions and for improving the frequency of parents reading to children (Mayer et al., 2019). *Status quo bias* can also interfere with changes in parent behavior, favoring inertia and defaults over steps that require action such as enrolling in a parenting program (DellaVigna, 2009). The *endowment effect bias* posits that individuals prefer and are less willing to give up items that they already own (Kahneman et al., 1991). The BE approach positions these factors alongside conventional economic factors in a decision-making framework. For example, providing information (conventional economic theory) is coupled with considering how such information can be presented to reduce stigma or attentional demands (BE theory).

The ParentCorps outreach period begins 5 weeks prior to session 1 of the parenting program, and personal outreach to families who have not yet attended (or who declined) continues until session 5. We designed a bundle of BE-infused outreach materials with the intention of delivering one ParentCorps-related communication per week to avoid saturation. All printed outreach materials were delivered to parents through their children’s backpacks. We designed the BE-informed outreach materials and an outreach plan/schedule to mirror the materials and level of effort used in the standard outreach practices (referred to as core outreach) (see Online Resource Figures A through E for images of materials). Thus, we hypothesized that implementation of the BE outreach would not require any more or less effort for site-level program staff and therefore would be as feasible as the standard outreach. Some additional effort was required by ParentCorps administrators to ensure that they delivered BE outreach materials to BE sites and standard outreach materials to control sites.

To address the specific psychological contexts identified as potential bottlenecks in the parenting program context, we infused BE concepts into the outreach materials, as shown in Fig. 1, level 3. The first communication shared with parents was the “Real Talk” *brochure insert* (addressing stigma, fear of judgment, negative attribution). The “Real Talk” insert contained the ParentCorps logo and the tagline “Together We: Parent. Share. Learn. Grow” to reduce the stigma that parents may perceive for being singled out as *needing* parenting support. The insert was also designed to reduce attentional demands with clear, concise, and visually appealing information about the program’s ethos that “*all* parents can benefit from parenting programs” and that parents bring expertise that can support other parents.

One week after the delivery of the “Real Talk” insert with the ParentCorps brochure, parents received a *letter from the Teacher and a wearable button* (addressing social norms and social identity. The personalized letter framed ParentCorps attendance as the social norm for pre-K parents at the school and included signatures (i.e., endorsements) of all the pre-K lead and assistant teachers at the site. The wearable button that included the name of the pre-K program, the ParentCorps logo, and “Proud Pre-K Parent” in large print to foster social and group identity around ParentCorps. Three weeks before the start of parenting program weekly sessions, families received an *affirmation postcard* (addressing stigma, fear of judgment, and social norms) that included a reflective prompt asking parents to think or write about a time unrelated to parenting when they felt successful or proud. Self-affirmations, developed in psychology and well tested in the schooling and health domains (Cohen & Sherman, 2014), have been shown to elicit positive self-concept. Indeed, a pride-based affirmation was found to increase parents’ positive self-concept and subsequent interest in parenting resources, especially among parents with high baseline fear of judgment and who had trouble meeting monthly expenses (Hill et al., 2020). The card also included the statement “Being a parent is full of joy and struggles” to help establish the norm that parenting entails highs and lows. The postcard contained the ParentCorps logo and tagline to facilitate association with the program.

Two weeks prior to the first parenting program meeting, families received *prize drawing tickets* (addressing present bias and endowment effect bias) for use at each session. These tickets were printed with the ParentCorps logo and packaged like a gift with cellophane wrapping and ribbon. The ticket provided parents with a tangible reminder of the potential for small financial incentives at sessions (BETA Project, 2013). In addition, providing the prize drawing tickets prior to sessions drew on the endowment effect bias for parents who might be less willing to lose the potential value of their tickets by not attending.

Families were invited with an *active choice invitation* to counter inertia or status quo bias (Castleman et al., 2019; Keller et al., 2011). The invitation was delivered via a card that asked “Will you attend ParentCorps on [date] at [time]?” The two options were “yes” or “no.” A sentence highlighting an advantage of attending was included next to the “yes” option (i.e., “Yes. I will attend ParentCorps to learn strategies to help my child succeed in school”).

In addition, to encourage attendance throughout the 14 sessions, we developed a suite of text messages as part of the BE outreach. The messages contained conventional reminders about session dates and times, tidbits of program content, and novel messages aimed at addressing psychological contexts likely to influence parents’ decision-making. With approval from the school district and agreement from the pre-K program directors, parents were automatically enrolled into receiving text messages, with the option to opt out. During the 14-week program cycle, all parents in the treatment sites who did not opt out of receiving text messages received one message per week.

### Methods for Randomized Testing of BE-Infused Outreach Components

The current study was initiated with 11-center-based pre-K programs in their first year of ParentCorps implementation in Brooklyn, the Bronx, and Queens in New York City. Within a larger hybrid effectiveness-implementation trial, six sites were randomized to the BE-infused outreach group and five sites to the core outreach group. Among the BE-infused sites, one site chose not to participate in the feasibility study and another site did not agree to the text messages; thus, the current study focuses on the four sites with the full set of BE-infused outreach strategies and the five sites with core outreach materials that launched the ParentCorps parenting program in winter 2018 (see Online Resource Table A). All sites received the same number of print materials, delivered at the same time points during outreach, which was once per week starting 5 weeks prior to the first session.

Within the BE sites, we implemented a family-level randomized sub-study in which half of the families received text messages at varying times (i.e., Mondays at 9:00 a.m. and Fridays at 2:00 p.m.), while the other half of families received text messages at the same time (i.e., Mondays and Fridays at 6:30 p.m.). Recognizing that people may habituate to receiving text messages and thus pay less attention over time, we delivered messages at varying times to boost novelty and to respect that parents’ schedules and interest in reading messages may vary at different times. Cunha et al. (2017) demonstrated success in varying the delivery time of text messages in the school context. We used the random number generator and sorting functions in Excel to randomize families into treatment (varying time) and control (consistent time) groups.

### Data and Sample

Through an administrative data request to the NYC Department of Education, we obtained descriptive pre-K site-level demographic characteristics for the 2017–2018 school year. The total number of children enrolled was obtained in winter 2018 with no adjustment for withdrawals during spring 2018, when the 14-week parenting program was delivered. Table 1 presents a descriptive profile of the nine sites, which included 621 unique families (238 families in the four BE-infused outreach sites and 383 families in the five core outreach sites). Sites ranged from serving 26 to 151 children. In most sites, more than 70% of families lived in census tracts with concentrated poverty (one site was an outlier at 29%). Descriptively, the percent of Latinx children ranged substantially across sites (from 16.0 to 90.5%). On average, BE-infused outreach sites were smaller than core outreach sites (59.6 vs. 76.6 children), had a lower percentage of Latinx students (an average of 51.5% vs. 63.7%), and comparable percentages of students in poverty (72.4% vs. 79.5%). Of the 238 families in the BE outreach sites, 220 were kept in the analytic sample because their families had cell phone numbers accurately matched to family attendance data.

**Table 1:** Analytic sample demographic characteristics

Group	Number of children enrolled	Percent Latinx	Percent in poverty
Full sample	621	56.4	73.0
BE-infused outreach	238		
Site 1	69	61.4	97.1
Site 2	31	16.0	80.0
Site 3	112	32.6	29.2
Site 4	26	95.8	83.3
Average	59.5	51.5	72.4
Core outreach	383		
Site 5	39	75.9	86.2
Site 6	66	90.5	73.8
Site 7	76	66.3	81.3
Site 8	151	58.2	83.6
Site 9	51	27.6	72.4
Average	76.6	63.7	79.5

Individual family-level characteristics are not available. Site-level characteristics are drawn from site administrative enrollment data. Enrollment data informing attendance calculations used an updated enrollment data set provided by sites in winter 2018

### Measures

Attendance is measured from data collected through a sign-in sheet displayed at the entrance to the room where the session was held. Facilitators also recorded the total number of individuals present. We measure attendance at the child level (any parent or caregiver for a given child) in three ways: (1) attendance at a single session between sessions 1 and 5 (this period was chosen because ParentCorps engages in outreach to families through session 5); (2) attendance at least once during the approximately 14-week program; and (3) percentage of sessions attended by those who attended at least once. We use a percentage instead of the raw number of sessions attended to adjust for cancellations due to weather or other logistical constraints.

### Analytic Approach

We examined unadjusted differences between the BE-infused outreach and core outreach sites and estimated adjusted differences via a regression-based approach that included controls for site-level percentage of families in poverty, site-level percentage of Latinx families, and total site enrollment. We include a control for site-level percentage of families in poverty because one of the premises underlying this work is that the context of poverty (i.e., financial constraint and low access to resources) drains individuals’ mental bandwidth that parents could otherwise direct toward learning about, considering, and perhaps attending the program. Therefore, we expect that poverty has suppressing or negative effects on program attendance. We also control for the site-level percentage of Latinx families because anti-Latinx sentiment, discrimination against immigrants, and fears of deportation that were particularly salient among Latinx communities during the period of this study (Robbins, 2016) may have had suppressing or negative effects on program attendance. Finally, we control for total site enrollment because sites with fewer families may be better able to engage in family outreach, and therefore, higher enrollment numbers may have a negative impact on program attendance.

For the sub-study of text message timing in the BE outreach group, we estimated a site fixed-effects model:$${Y}_{\mathrm{i}}= {\beta }_{0}+{\beta }_{1}{(varying text message time)}_{\mathrm{i}}+{\delta }_{\mathrm{n}}+{\varepsilon }_{\mathrm{i}}$$ with the parameter of interest as $${\beta }_{1}$$ interpreted as the change in probability of attending a given session or ever attending a session, or as the change in the percentage of sessions attended for those who received varying-time messages. The parameter $${\delta }_{\mathrm{n}}$$ is a site fixed effect, and $${\varepsilon }_{\mathrm{i}}$$ is an error term.

## Results

There was substantial descriptive variation in attendance across the core and BE-infused outreach sites (see Online Resource Table B). The percentage of families that ever attended varied from 12% (at site 3, a BE-infused outreach site) to 73% (at site 9, a core outreach site). Among families who ever attended, the average percentage of sessions attended varied from 23% (at site 9, a core outreach site) to 60% of sessions attended (at site 2, a BE site). Among all sites (see Online Resource Table C), attendance was highest at session 1, with 19% of all families attending, and decreased thereafter, with 11% of families attending session 5. In the core outreach group, 37% of families ever attended, whereas 30% of families in the BE-infused outreach group ever attended. However, those in the BE-infused outreach group attended a higher percentage of sessions than those in the core outreach group (38% vs. 33%).

As shown in Table 2, the site-level randomized implementation of BE-infused outreach materials did not show a statistically significant impact on attendance. Families at BE-infused outreach sites had a higher predicted probability of attending session 2 (*b* = 0.064, se = 0.04, *p* = 0.185), session 4 (*b* = 0.000, se = 0.02, *p* = 0.994), and session 5 (*b* = 0.005, se = 0.04, *p* = 0.904) and a lower predicted probability of attending session 1 (*b* =  − 0.031, se = 0.06, *p* = 0.605) and session 3 (*b* =  − 0.038, se = 0.04, *p* = 0.402); however, these estimates do not meet the threshold of statistical significance at *p* < 0.05. As we expected, larger sites (i.e., total pre-K enrollment) had lower rates of attending any session between sessions 1 and 5 and ever attending.

**Table 2:** The impact of BE-infused outreach on attendance

Variable	Session 1	Session 2	Session 3	Session 4	Session 5	Ever attend	Percent of sessions attended
BE-infused outreach	−0.031	0.064	−0.038	0.000	0.005	−0.129	0.072
	(0.06)	(0.04)	(0.04)	(0.02)	(0.04)	(0.09)	(0.07)
Percent of families in poverty at site	0.040	0.073	0.046	0.030	−0.010	0.186	−0.569*
	(0.11)	(0.12)	(0.11)	(0.06)	(0.10)	(0.17)	(0.16)
Percent of Latinx families at site	0.135	0.202	0.005	0.059	0.047	−0.128	0.183
	(0.20)	(0.18)	(0.17)	(0.07)	(0.15)	(0.21)	(0.20)
Site enrollment	−0.002*	−0.001*	−0.002*	−0.001*	−0.002*	−0.004*	−0.001
	(0.00)	(0.00)	(0.00)	(0.00)	(0.00)	(0.00)	(0.00)
Constant	0.258**	0.071	0.275*	0.158*	0.226*	0.703*	0.719*
	(0.13)	(0.09)	(0.10)	(0.04)	(0.08)	(0.21)	(0.17)
Number of observations	621	621	621	621	621	621	215

Standard errors are in parentheses*. Ever attend* is a dichotomous variable for whether a family attended at least once in the 14-week program

*BE* behavioral economics

**p < .1; *p < .05

The sub-study showed positive impacts of time-varying text messages on families’ attendance across two attendance metrics (Table 3). In the BE-infused outreach sites, families randomized to receive messages at varying times were more likely to ever attend the program (*b* = 0.111, se = 0.06, *p* < 0.05), compared with families at the same site who received messages at a consistent time. This difference was significant for session 2 attendance as well (*b* = 0.102, se = 0.05, *p* < 0.05), which corresponds with the onset of text messaging.

**Table 3:** The impact of varying time text messages on attendance among families in the BE-infused outreach sites

Variable	Session 1	Session 2	Session 3	Session 4	Session 5	Ever attend	Percent of sessions attended
Varying time	0.030	0.102*	0.058	0.057	0.021	0.111*	− 0.005
	(0.05)	(0.05)	(0.04)	(0.04)	(0.04)	(0.06)	(0.08)
Consistent time	0.153**	0.108**	0.089**	0.099**	0.117**	0.235**	0.380**
	(0.03)	(0.03)	(0.03)	(0.03)	(0.03)	(0.04)	(0.06)
Observations	220	220	220	220	220	220	64

*Ever attend* is a dichotomous variable for whether a family attended at least once in the 14-week program. Percentage of sessions attended is calculated based on the total number of sessions a family attended out of the 14-week series (or the total number of sessions delivered at a site in the event of cancellation due to weather or other scheduling challenges); estimates for the percentage of sessions attended include only families who attended at least once

*p < .05; **p < .01

## Discussion

This study describes the contribution of BE for optimizing participation in parenting programs through a unique collaboration between developers of an evidence-based parenting program, the team supporting implementation at scale, and researchers with expertise in BE. We used existing research on parent engagement and interviews and focus groups with ParentCorps parents, developers, and coaches to inform our identification of relevant BE concepts, such as drain of cognitive resources, stigma, fear of judgment, negative attribution, social norms, social identity, present bias, status quo bias, and endowment effect bias. These concepts in turn informed our development of BE-infused print and digital outreach materials tailored to the specific context of the ParentCorps parenting program. The BE-infused bundle included a “Real Talk” brochure insert, a teacher letter and wearable button, a postcard self-affirmation reflection, an active choice invitation to participate in the parenting program, and a suite of text-based messages. These BE-infused outreach materials were collaboratively designed with program developers and implementers aiming to minimize operational or varying labor and material costs. The BE-infused bundle was successfully implemented by pre-K staff, meaning that all BE outreach materials were distributed to program sites and subsequently distributed to families consistent with the standard outreach schedule, thus demonstrating feasibility in the context of scaling up efforts to serve families in historically disinvested neighborhoods. The ParentCorps program continues to use most of the BE-infused printed materials (i.e., the Real Talk insert, teacher letter, affirmation postcard, and active choice invitation) as part of their outreach. The prize drawing tickets were discontinued due to district-level challenges in funding prizes.

We utilized an iterative process to translate BE concepts into outreach materials that incorporated input from parents and practitioners consistent with the Rapid Synthesis and Translation Process (Thigpen et al., 2012). The design of the active choice invitation is illustrative of how our team collaboratively addressed challenges. We began from the idea of addressing inertia in decision-making by shifting the default from opt-in to opt-out, as is typical in BE research. This was initially operationalized as a ParentCorps membership card, given to all families at the pre-K site, along with a session date/time assignment as a strategy to signal enrollment without requiring any action from parents. Through review, we understood that this approach may not align well with principles of preserving autonomy, fostering engagement beyond initiation, and doing no harm. We also did not want default choices to be misinterpreted as parents falling short. Instead, we implemented an enhanced active choice invitation with an explicit option to choose “yes” or “no” that highlighted the benefits of attending sessions, avoiding the suggestion that non-attendance was an example of falling short as a parent.

With respect to our second aim—to report on the effect of testable components of the BE-infused outreach strategies on parents’ participation—our site-level randomization was appropriate for meeting our first aim but with a small sample size (*n* = 9) and low power, was not optimal for testing the impact of BE-infused materials on parent attendance. There were no statistically significant differences between attendance at BE-infused and core outreach sites. Descriptive data show substantial variation in attendance within core and BE-infused outreach sites. However, from the available site-level descriptive data, we are unable to determine factors that contributed to this variation.

In contrast, our embedded family-level randomization sub-study demonstrated that varying the time at which parents receive text messages every week—an insight around novelty of information as influencing behavior—improved attendance at session 2, which followed the onset of text messaging, and improved the overall likelihood of families ever attending, compared with families that received text messages at a consistent time.

We hypothesized that varying message delivery times capitalizes on two features of how families make decisions. First, receiving text messages at unpredictable times might increase the novelty of the communication and thus better draw attention to the messages, and, second, alternating the time of text message delivery increases the likelihood that any one message lands in a “viewing window” when parents may be attentive. Digital forms of outreach are increasingly popular methods for reaching children’s caregivers and are largely consistent with BE principles of light touch, low-cost, and streamlined approaches. We built on this by infusing BE thinking in message content, timing, and specifics (e.g., personalization). Less than 5% of the parents who received messages chose to opt out. However, text messages and related digital-based methods of communication come with trade-offs, particularly in the context of relationship-based programs implemented at scale. There are enormous labor costs of responding to messages if parents view them as modes of feedback or confirmation, with lack of response potentially reducing trust. As many more pre-K programs now communicate with families through text messaging and digital platforms, ParentCorps is working with programs to use BE-infused content in their messaging and varying the message timing based on the current findings.

This study relied on existing program data to measure outcomes, and thus, research costs were primarily to cover the cost of staff for conceptual work, design, and analysis, not data collection. Apart from the fixed costs of creating the reusable suite of BE text messages, a back-of-the-envelope calculation applied in a previous study finds that the per-family additional cost of BE-infused print and digital outreach was below $1.00, substantively lower than the approximate cost of $6.25 per family for motivational interviewing (Winslow et al., 2016).

### Limitations

This study has several limitations. First, our site-level randomization design was useful in assessing the feasibility of implementing a BE outreach strategy that mirrored the standard outreach strategy, but the design was not optimal for testing the impact of BE-infused outreach on parent attendance. Future research could consider site-level randomization with a larger number of sites, or utilize family-level randomization for tests in ways that are acceptable to pre-K directors (in the current sub-study, all families received the same message content but at different times). Second, BE sites received a bundle of BE-infused outreach materials, so we are unable to determine the efficacy of any singular piece of BE-infused outreach material or the efficacy of addressing one bias compared to another. Future research can address this challenge by developing more narrowly tailored approaches to measuring the impact of specific bias-addressing materials. Third, we did not examine outcomes beyond program attendance; therefore, we are unable to assess whether the BE-infused outreach materials had impacts on application of program strategies at home, or involvement in children’s learning. Fourth, while this study is rooted in the context of efforts to scale up a parenting program, our work focused on a selected set of newly implementing sites as opposed to all system sites operating at full scale. Thus, the implications for attendance or other measures of family engagement from cross-fertilization and spillover that may occur with a universally scaled approach might differ.

### Implications

Optimizing parent attendance in evidence-based programs that support the well-being of children remains challenging, especially at scale in public systems (Al-Ucayali et al., 2019; Fagan et al., 2019). BE offers a framework that can be applied to support parent engagement during program development and evaluation phases, including efficacy and effectiveness testing and full-scale implementation. Grounded in the cost–benefit decision-making tools of conventional economics, BE considers how in-the-moment decision-making can be shaped by social-psychological factors and can disrupt or facilitate the interconnected sequence of decisions that affect observed parenting choices and behavior. By turning a lens on circumstances and the role of cognitive resources, the BE framework incorporates how the context of poverty can especially drain attention and interfere with follow-through despite intentions.

As evidenced by our collaborative process in this study, BE offers insights on how to enhance outreach and implementation in low-cost ways that are easily integrated into existing systems and processes. However, researchers and program administrators will need to invest time and resources into understanding the contexts that may be impacting individuals’ behavior and decision-making in specific contexts, and into the iterative process of developing and testing program-consistent materials that address the identified socio-psychological biases.

## Supplementary Information

Below is the link to the electronic supplementary material.

Supplementary file1 (DOCX 1552 KB)
