# Supplementary material for: Behavioral Economics and Parent Participation in an Evidence-Based Parenting Program at Scale
Source: Prev Sci. 2021 May 20;22(7):891–902. doi: 10.1007/s11121-021-01249-0 (PMC8458200; doi:10.1007/s11121-021-01249-0)
Supplement: Supplementary file 1 — Supplementary file1 (DOCX 1552 KB) [file 11121_2021_1249_MOESM1_ESM.docx]

Supplement

| **Table A. Comparison of core and BE-Infused Outreach groups** | | |
| --- | --- | --- |
| **Timeline** | **BE-Infused Outreach** | **Core Outreach** |
| *5 weeks before* | Brochure with "Real Talk" Brochure Insert | Brochure with "Coming Soon" Insert |
| *4 weeks before* | Letter from Teacher with Button | Flyer with program information |
| *3 weeks before* | Affirmation Postcard | Flyer with program date/time |
| *2 weeks before* | Prize Drawing Tickets | Flyer with sign-up sheet |
| *1 week before* | Active Choice Invitation | Flyer with program information |
| *After session 1, until end of program* | Text messages outreach (with individual randomization of delivery time) | Reminder calls |
| *Note.* Timeline is in reference to the start date of session 1; for descriptions of BE-Infused Outreach, see *Current Study* section in manuscript. In both groups, teachers or family support staff attempted to engage individually with every parent in person or by phone; outreach continued until session 5 for any families who had not yet been reached. See Figures A through E for images of BE-Infused Outreach materials. | | |

| **Table B. Descriptive statistics of attendance by site** | | | |  |  |  |  |  |  |  |  |
| --- | --- | --- | --- | --- | --- | --- | --- | --- | --- | --- | --- |
|  |  |  |  |  |  |  |  |  |  |  |  |
|  | **BE-Infused Outreach Sites** | | | |  | **Core Outreach Sites** | | | | |  |
|  | *Site 1* | *Site 2* | *Site 3* | *Site 4* |  | *Site 5* | *Site 6* | *Site 7* | *Site 8* | *Site 9* |  |
|  |  |  |  |  |  |  |  |  |  |  |  |
| Attendance at session 1 | 0.23 | 0.39 | 0.08 | 0.15 |  | 0.33 | 0.41 | 0.21 | 0.10 | 0.14 |  |
|  |  |  |  |  |  |  |  |  |  |  |  |
| Attendance at session 2 | 0.25 | 0.32 | 0.06 | 0.23 |  | 0.15 | 0.36 | 0.11 | 0.05 | 0.08 |  |
|  |  |  |  |  |  |  |  |  |  |  |  |
| Attendance at session 3 | 0.13 | 0.39 | 0.04 | 0.15 |  | 0.23 | 0.30 | 0.11 | 0.05 | 0.16 |  |
|  |  |  |  |  |  |  |  |  |  |  |  |
| Attendance at session 4 | 0.14 | 0.23 | 0.06 | 0.19 |  | 0.13 | 0.23 | 0.12 | 0.07 | 0.14 |  |
|  |  |  |  |  |  |  |  |  |  |  |  |
| Attendance at session 5 | 0.13 | 0.32 | 0.06 | 0.15 |  | 0.15 | 0.27 | 0.07 | 0.03 | 0.12 |  |
|  |  |  |  |  |  |  |  |  |  |  |  |
| Ever attended | 0.49 | 0.48 | 0.12 | 0.38 |  | 0.46 | 0.61 | 0.33 | 0.15 | 0.73 |  |
|  |  |  |  |  |  |  |  |  |  |  |  |
| Percent of sessions attended^a^ | 0.24 | 0.60 | 0.53 | 0.33 |  | 0.31 | 0.51 | 0.25 | 0.26 | 0.23 |  |
|  | (0.23) | (0.30) | (0.32) | (0.35) |  | (0.28) | (0.33) | (0.28) | (0.28) | (0.23) |  |
|  |  |  |  |  |  |  |  |  |  |  |  |
| Observations | 69 | 31 | 112 | 26 |  | 39 | 66 | 76 | 151 | 51 |  |
| Note. Coefficients are means of all pre-K parents in attendance at a given session or ever attended; | | | | | | | | |  |  |  |
| ^a^Percent of sessions attended is out of the total number of sessions offered at the site, adjusted to include only those who ever attended | | | | | | | | | | | |

| **Table C. Descriptive statistics of attendance by outreach group** | | | | | | | | | | | |
| --- | --- | --- | --- | --- | --- | --- | --- | --- | --- | --- | --- |
|  |  |  |  |  |  |  |  |  |  |  |  |
|  | **Whole Sample** | |  | **Core Outreach** | |  | **BE-infused Outreach** | |  | **Difference in means** | |
|  | *mean* | *sd* |  | *mean* | *sd* |  | *mean* | *sd* |  | *b* | *t* |
| Attendance at session 1 | 0.19 | 0.39 |  | 0.20 | 0.40 |  | 0.17 | 0.38 |  | 0.03 | 0.97 |
| Attendance at session 2 | 0.14 | 0.35 |  | 0.13 | 0.34 |  | 0.17 | 0.37 |  | -0.04 | -1.29 |
| Attendance at session 3 | 0.13 | 0.34 |  | 0.14 | 0.35 |  | 0.12 | 0.33 |  | 0.02 | 0.59 |
| Attendance at session 4 | 0.12 | 0.33 |  | 0.12 | 0.33 |  | 0.12 | 0.33 |  | -0.00 | -0.06 |
| Attendance at session 5 | 0.11 | 0.31 |  | 0.10 | 0.30 |  | 0.13 | 0.33 |  | -0.02 | -0.93 |
| Ever attended | 0.35 | 0.48 |  | 0.37 | 0.48 |  | 0.30 | 0.46 |  | 0.07 | 1.81 |
| Percent of sessions attended | 0.35 | 0.31 |  | 0.33 | 0.30 |  | 0.38 | 0.31 |  | -0.05 | -1.17 |
| Observations | 621 |  |  | 383 |  |  | 238 |  |  | 621 |  |
| *Note.* Percent of sessions attended is out of the total number of sessions offered at the site. | | | | | | | | |  |  |  |

**Figure A. BE-Infused Outreach Material: Letter from Teacher with Button**

*Note.* BE-Infused Outreach material sent to families 5 weeks before the start of session 1.

**Figure B. BE-Infused Outreach Material: “Real-Talk” Brochure Insert**


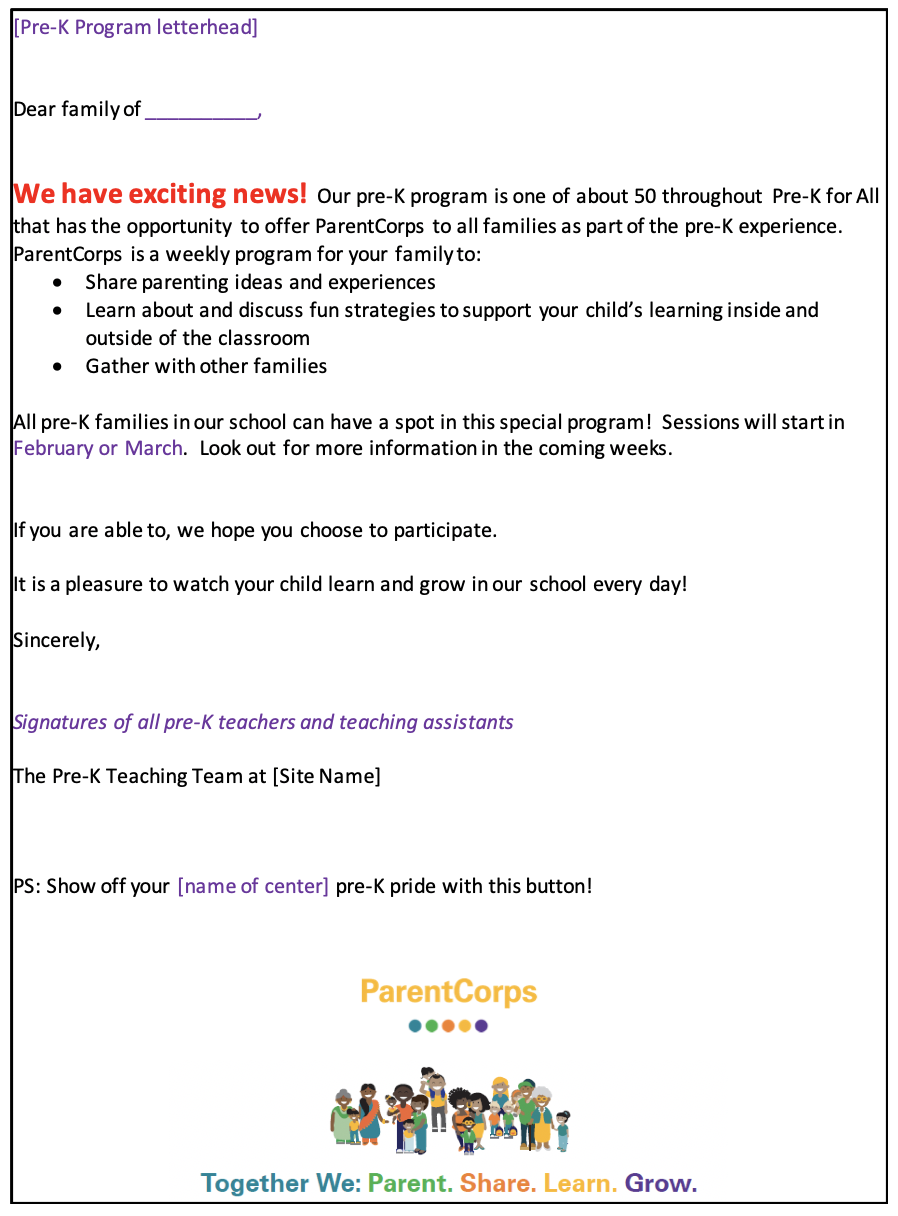


[Name of]

*Note.* BE-Infused Outreach material sent to families 4 weeks before the start of session 1.

**Figure C. BE-Infused Outreach Material: Affirmation Postcard**

*Note.* BE-Infused Outreach material sent to families 3 weeks before the start of session 1.

**Figure D. BE-Infused Outreach Material: Prize Drawing Tickets**


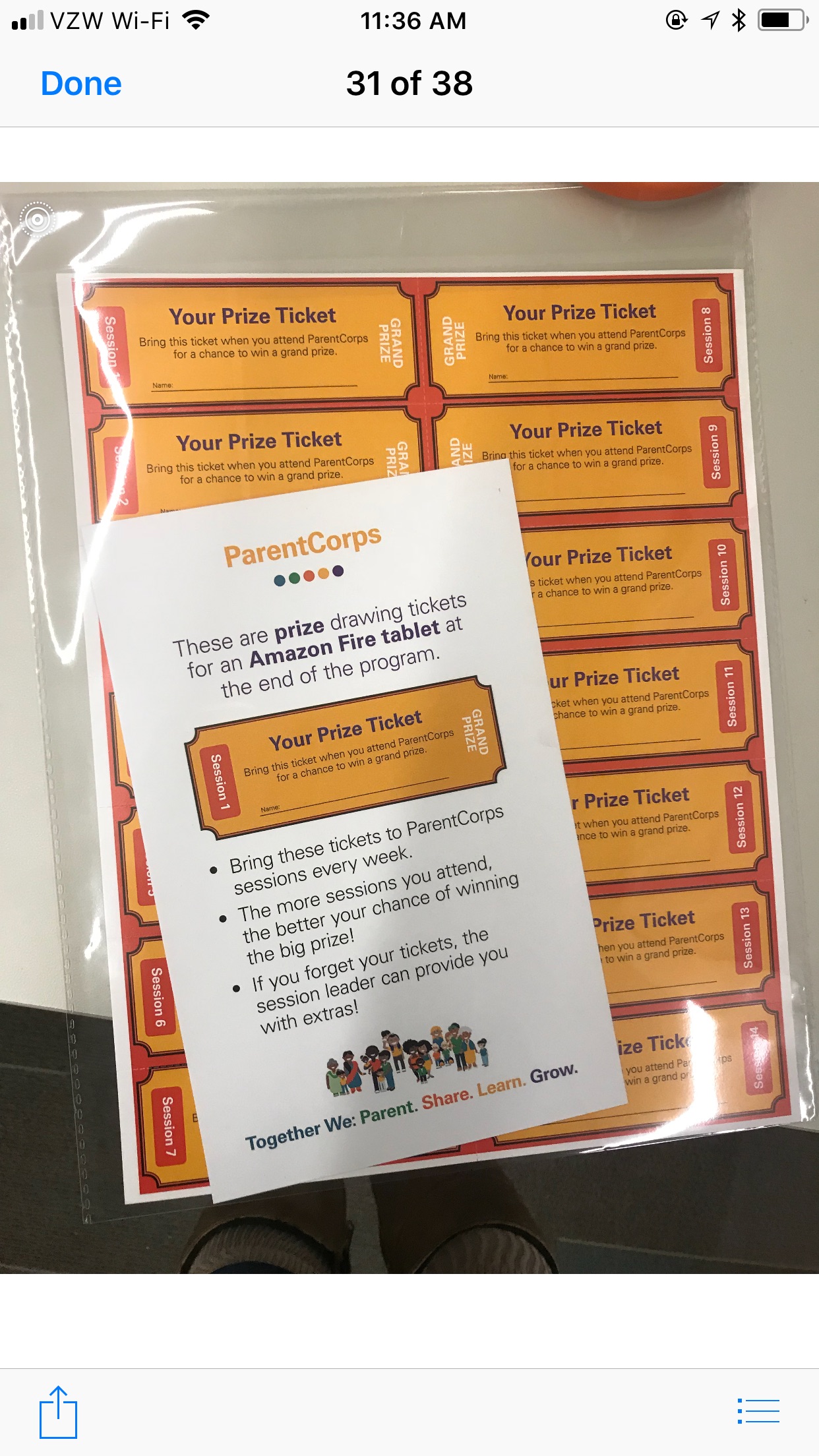


*Note.* BE-Infused Outreach material sent to families 2 weeks before the start of session 1.

**Figure E. BE-Infused Outreach Material: Active Choice Invitation**

*Note.* BE-Infused Outreach material sent to families 1 week before the start of session 1.
